# Expression of Active Subunit of Nitrogenase via Integration into Plant Organelle Genome

**DOI:** 10.1371/journal.pone.0160951

**Published:** 2016-08-16

**Authors:** Natalia B. Ivleva, Jeanna Groat, Jeffrey M. Staub, Michael Stephens

**Affiliations:** Monsanto Company, 700 Chesterfield Village Parkway, Chesterfield, MO, United States of America; International Centre for Genetic Engineering and Biotechnology, INDIA

## Abstract

Nitrogen availability is crucial for crop yield with nitrogen fertilizer accounting for a large percentage of farmers’ expenses. However, an untimely or excessive application of fertilizer can increase risks of negative environmental effects. These factors, along with the environmental and energy costs of synthesizing nitrogen fertilizer, led us to seek out novel biotechnology-driven approaches to supply nitrogen to plants. The strategy we focused on involves transgenic expression of nitrogenase, a bacterial multi-subunit enzyme that can capture atmospheric nitrogen. Here we report expression of the active Fe subunit of nitrogenase via integration into the tobacco plastid genome of bacterial gene sequences modified for expression in plastid. Our study suggests that it will be possible to engineer plants that are able to produce their own nitrogen fertilizer by expressing nitrogenase genes in plant plastids.

## Introduction

Crop yield derives from a complex interaction of multiple genetic and environmental factors[1, 2]. A crucial component of any crop production is the presence of sufficient amounts of nitrogen[3, 4]. Particularly, nitrogen is critical for maximizing yields in commercial crops[5, 6]. To ensure adequate crop performance, farmers have been continuously increasing application of nitrogen fertilizer throughout the world, and nitrogen fertilizer has been accounting for a large percentage of farming expenses[7, 8]. However, it is also known that excessive or untimely application of nitrogen fertilizer can have a negative impact on the environment[4]. Nitrogen fertilizer is a major source of reactive nitrogen in water and air, and is considered to be a key contributor to global climate change[9]. In addition, the production of nitrogen fertilizer is energy intensive, further contributing to climate change. As described below, modern biotechnology might be uniquely positioned to ensure nitrogen supply to plants without the use of synthetic nitrogen fertilizer and, therefore, would provide benefits to farmers while reducing the risks of negative environmental effects.

Despite the fact that nitrogen is the most prevalent element in the Earth’s atmosphere and accounts for about 80% of the air, atmospheric nitrogen is not readily available for plants. Only a few prokaryotic species are able to convert (“fix”) atmospheric nitrogen to chemical compounds which plants can use as a source of nitrogen[10, 11]. This nitrogen fixation process is performed by nitrogenase, a highly conserved enzyme present in all bacterial species that are able to fix nitrogen. Several forms of nitrogenase have been characterized, with molybdenum-dependent nitrogenases being the best-studied group of nitrogenases[10]. Molybdenum-dependent nitrogenase is a complex enzyme that consists of 2 components: the Fe protein which is encoded by the *nifH* gene, and the MoFe protein which is encoded by the *nifD* and *nifK* genes. The Fe protein (also known as dinitrogenase reductase) is the obligatory donor of electrons to the MoFe protein (also known as dinitrogenase) which reduces atmospheric nitrogen to ammonia. In addition to the structural proteins encoded by *nifH*, *nifD* and *nifK* genes, the active enzyme requires highly specialized chaperons and a set of unique iron and molybdenum clusters, the biosynthesis of which is considered to be one of the most complex in nature, and requires additional (up to 17) unique proteins[12].

Since the dawn of biotechnology, scientists have been discussing the possibility of creating plants that could synthesize their own nitrogen fertilizer[13, 14]. Currently, three major approaches are considered for engineering of nitrogen-fixing plants[7, 15, 16]. The first approach is focused on engineering of symbiotic interactions between plants and nitrogen-fixing bacteria, which would imitate (and possibly improve) the nodule formation currently existing in soybeans and other legumes[17]. The second approach looks at the possibility of introducing and/or improving the nitrogen- fixing pathway in bacterial endophytes that are already known to have symbiotic relationships with plants[15, 18]. The third approach (which is the focus of this report) targets the incorporation of bacterial nitrogenase genes into the plant genome and, therefore, would enable the plant itself to produce the nitrogen-fixing enzyme and fix the atmospheric nitrogen. All approaches mentioned above are expected to be challenging to execute due to the complexity of plant-microbe interactions (in the case of approaches 1 and 2 described above), the complexity of nitrogenase biogenesis, and the oxygen sensitivity of the enzyme (see below)[7, 16].

The first report of transgenic production of nitrogenase was published in the 1970s, when large fragments of chromosomal DNA containing the *nif* cluster were transferred via conjugation from nitrogen-fixing bacteria *Klebsiella pneumoniae* into *Escherichia coli*, a species which does not naturally fix nitrogen[19]. The resulting strain was able to capture atmospheric nitrogen. In the past several years work on transgenic nitrogenase production has accelerated and resulted in the engineering of multiple bacterial strains that are able to produce the functional transgenic nitrogenase[20–22]. However, the production of nitrogenase in eukaryotes, including plants, has still not been accomplished[7]. The expression of nitrogenase in plant tissue faces two major challenges. First, as mentioned above, the production of active nitrogenase is very complex[12] and requires expression of at least 9 genes in bacteria[21] and possibly even more in eukaryotic organisms. It is also believed that the *nif* genes need to be expressed in a certain ratio[23–25], and the precise expression of such a large number of transgenes is not trivial in plants. The second challenge is that the nitrogenase enzyme is extremely sensitive to oxygen and is irreversibly inactivated after exposure to air[26]. Since plants are a major source of oxygen production in the biosphere, additional optimization of nitrogenase expression (such as expression at night or in non-photosynthetic tissues) may be needed.

The expression of a functional Fe protein in plant tissue could serve as a crucial step in improving our understanding of the limitations and opportunities for expressing a functional nitrogenase in plants. Out of the two components (subunits) required for functional nitrogenase, the Fe protein is the simpler one as it consists of a single structural protein (NifH) and contains a relatively simple iron-sulfur cluster[7]. NifM protein is required for the maturation of NifH[27]. Importantly, the Fe protein not only plays an enzymatic role as dinitrogenase reductase but is also needed for the assembly of the MoFe protein. In the absence of transgenic MoFe, the activity of the plant-produced Fe protein could be assayed *in vitro* when combined with a MoFe protein isolated from a bacterial source. It is known that the Fe protein is the more oxygen sensitive (out of two) component of the nitrogenase complex[26], suggesting that, if an active Fe subunit could be produced in certain tissues and/or under certain growth conditions, these tissues and growth conditions could also produce active nitrogenase. Previously, it was reported that proteins required for the Fe subunit were produced in tobacco leaf and in *Chlamydomonas reinhardtii* cells[28, 29]. Though plant-produced Fe protein was not able to complement bacteria-produced MoFe protein, *nif* transgenes were able to complement a *Chlamydomonas* mutation in a gene encoding a chlorophyll biosynthesis enzyme similar in function to the Fe protein[28]. This complementation indicated that at least some functionality was retained in the algae-produced Fe protein, although dinitrogenase reductase activity was not demonstrated.

Here we report the transgenic production of the active Fe protein of nitrogenase in a higher plant species. We demonstrate that genes *nifH* and *nifM* were integrated into the tobacco chloroplast genome, and the resulting tobacco plants produced NifH and NifM proteins. The functional activity of the plant-produced NifH protein was demonstrated *in vitro* by combining bacteria-produced MoFe protein with the protein fraction from the NifH-containing transgenic plants incubated transiently at low oxygen concentration. This study demonstrates that it is possible to engineer plants that produce the oxygen-sensitive components of nitrogenase, creating the first step in engineering an active nitrogenase enzyme in plants. Our early evaluation of plants grown under ambient oxygen concentration indicates that further optimization is needed for expression of an active Fe protein under these conditions, and additional studies are required for production of commercially viable nitrogen-fixing crops.

## Results

### Generation of transplastomic tobacco plants containing *nif* genes

**Fig 1:**
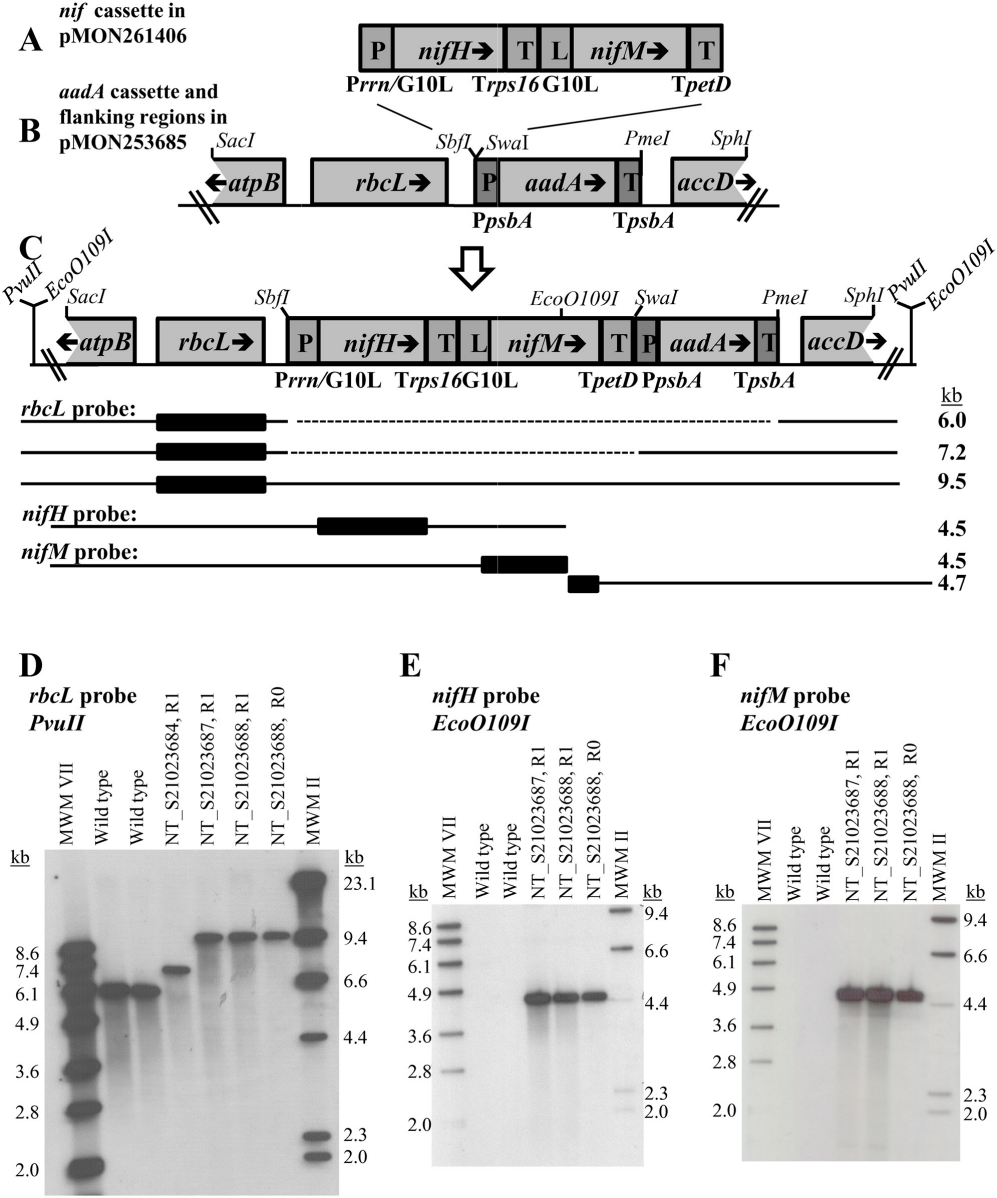
Transgenes *nifH* and *nifM* are integrated into the plastid genome, and transplastomic lines are homoplastomic. Restriction sites discussed in the manuscript are shown. (A) *nif* expression cassette from pMON261406. The vector contains a chimeric two-gene *nifH* and *nifM* operon expression cassette (*Prrn*:*G10L*:*nifH*:*Trps16*:*G10L*:*nifM*:*TpetD*). This two-gene operon cassette is cloned upstream of the selectable *aadA* gene cassette in pMON253685 (Fig 1B) to create plasmid pMON261406 (Fig 1C). (B) pMON253685 contains the the *aadA* gene expression cassette (*PpsbA*:*aadA*:*TpsbA*). This expression cassette is flanked by regions of homology to the endogenous plastid *rbcL* gene region (*SacI-SbfI* fragment) and to the endogenous plastid *accD* region (*PmeI-SphI* fragment). The vector pMON253685 is similar to pMON261406 except it lacks the chimeric *nifH* and *nifM* expression cassette. (C)The structure of the transplastomic lines NT_S21023687 and NT_S21023688 (produced as a result of the plastid transformation with pMON261406). Restriction enzyme sites used for Southern blot analysis (*PvuII* and *EcoO109I*) are located inside and outside of transforming DNA, which is delineated by *SacI* and *SphI* restriction enzyme sites. Black boxes represent hybridizing regions of the transplastomic genome; solid lines represent DNA fragments expected to hybridize with gene specific probes (not to scale). Indicated on the right are corresponding sizes (in kb) of the expected DNA fragments from Southern blot analysis that are shown in Figs D-E. DNA fragments and hybridizing regions corresponding to gemonic DNA of wild-type plants (6.0 kb) and the negative control event NT_S21023684 (7.2 kb) are also shown (D-F) Southern blot analyses of wild type and transplastomic lines. Tobacco genomic DNA was extracted from the wild type and transplastomic plants and digested with *PvuII* or *EcoO109I* restriction endonuclease. The DNA were separated on an agarose gel, transferred to a nylon membrane and hybridized with a digoxigenin-labeled probe specific to the *rbcL* gene (that is located close to the expected insertion site of the transgenes), *nifH* or *nifM* genes as indicated. Digoxigenin-labeled MWMVII and MWMII (Roche) indicate molecular weight markers, and sizes (kb) of MWM bands are indicated. The NT_S21023684 line (derived from the plastid transformation of pMON253685 and lacking *nif* genes) was included in the analysis as appropriate. (D) Southern blot of wild-type and transplastomic lines (R0 and R1 generations) after *PvuII* restriction enzyme digestion and hybridization with the resident *rbcL* coding region probe. Absence of wild type bands in the transplastomic samples indicates that these lines are homoplasmic for the inserted transgenic cassettes. (E) Southern blot of wild type and transplastomic lines after *EcoO109I* restriction enzyme digestion and hybridization with the *nifH* coding region probe. (F) Southern blot of wild type and transplastomic lines after *EcoO109I* restriction enzyme digestion and hybridization with the *nifM* coding region probe. For Figs E and F, note the expected absence of hybridizing bands in wild type samples.

To express the NifH and NifM proteins in plant tissue, DNA sequences encoding the *nifH* and *nifM* coding regions from *Azotobacter vinelandii* (Fig 1A) were engineered into a plastid transformation vector pMON253685 (Fig 1B). The resulting plasmid pMON261406 was designed to integrate the transgenes via homologous recombination downstream of *rbcL* gene of the tobacco plastid genome (Fig 1C). This integration site was previously reported to be used successfully for plastid transformations[30]. The construct pMON261406 was designed to constitutively express NifH and NifM proteins in plastids using a two-gene operon expression cassette. Multigene operons have previously been shown to be efficiently expressed in plastids[31], and this operon-based approach was chosen here to minimize the number of regulatory elements required for transgene expression. Transcription of the operon was driven by the strong constitutive ribosomal RNA promoter *Prrn*[32], and efficient translation of both transgenes was conferred by the bacteriophage T7 gene 10 leader *G10L*[32]. To stabilize transcripts, 3’-ends from tobacco plastid genes rps16[33] and petD (J. Staub, unpublished) were used. A chimeric *aadA* gene that confers spectinomycin antibiotic resistance[30] was used for selection of plastid transformants. Plasmid pMON261406 carries the *aadA* gene expression cassette downstream of the *nif* operon. Plasmid pMON253685 containing the *aadA* expression cassette (and not containing any *nif* genes) was used as a negative control in the experiments below. Tobacco leaves were transformed with pMON261406 and pMON253685 via biolistic bombardment, and spectinomycin resistant transplastomic lines were recovered in tissue culture after plant regeneration steps according to published protocols[30, 34].

The two transplastomic lines (NT_S21023687 and NT_S21023688) produced as a result of the plastid transformation with pMON261406 were analyzed by Southern blot analysis for the presence of the expected DNA inserts. Probes specific for the *nifH* and *nifM* coding regions were used (Fig 1C).The expected bands for the intact insertion of both *nif* genes were detected (Fig 1E and 1F; 4.5 kb for *nifH* and 4.5 and 4.7 kB for *nifM*) confirming the presence of both genes in the targeted integration site. Fig 1D shows a Southern blot analysis with a probe specific for the chloroplast *rbcL* gene that is located close to the expected insertion site of the transgenes. A sample from the wild type plants showed a DNA band corresponding to the expected uninterrupted wild-type region downstream of the plastid *rbcL* gene (6.0 kb). The line NT_S21023684, which derived from the plastid transformation using vector pMON253685 and contained the *aadA* gene but did not contain any *nif* genes, showed the expected larger DNA band (7.2 kb). Plants containing the *nifH* and *nifM* insert produced the expected 9.5 kb band (lines NT_S21023687 and NT_S21023688). As expected, all transplastomic lines lacked the lower molecular weight band corresponding to the wild type region in the absence of the insert (6.1 kb). These analyses indicated that the insertion of *nifH* and *nifM* genes were in the expected site of the chloroplast genome DNA and that the lines were homoplastomic (*i*.*e*. all chloroplast genome copies contained the desired insertion). R0 and R1 plants from one of the lines (NT_S21023688) were included into the analysis, and their Southern blot analysis indicated that the insertion was stable among progeny (Fig 1D–1F).

### Expression of plant-produced Nif proteins

**Fig 2:**
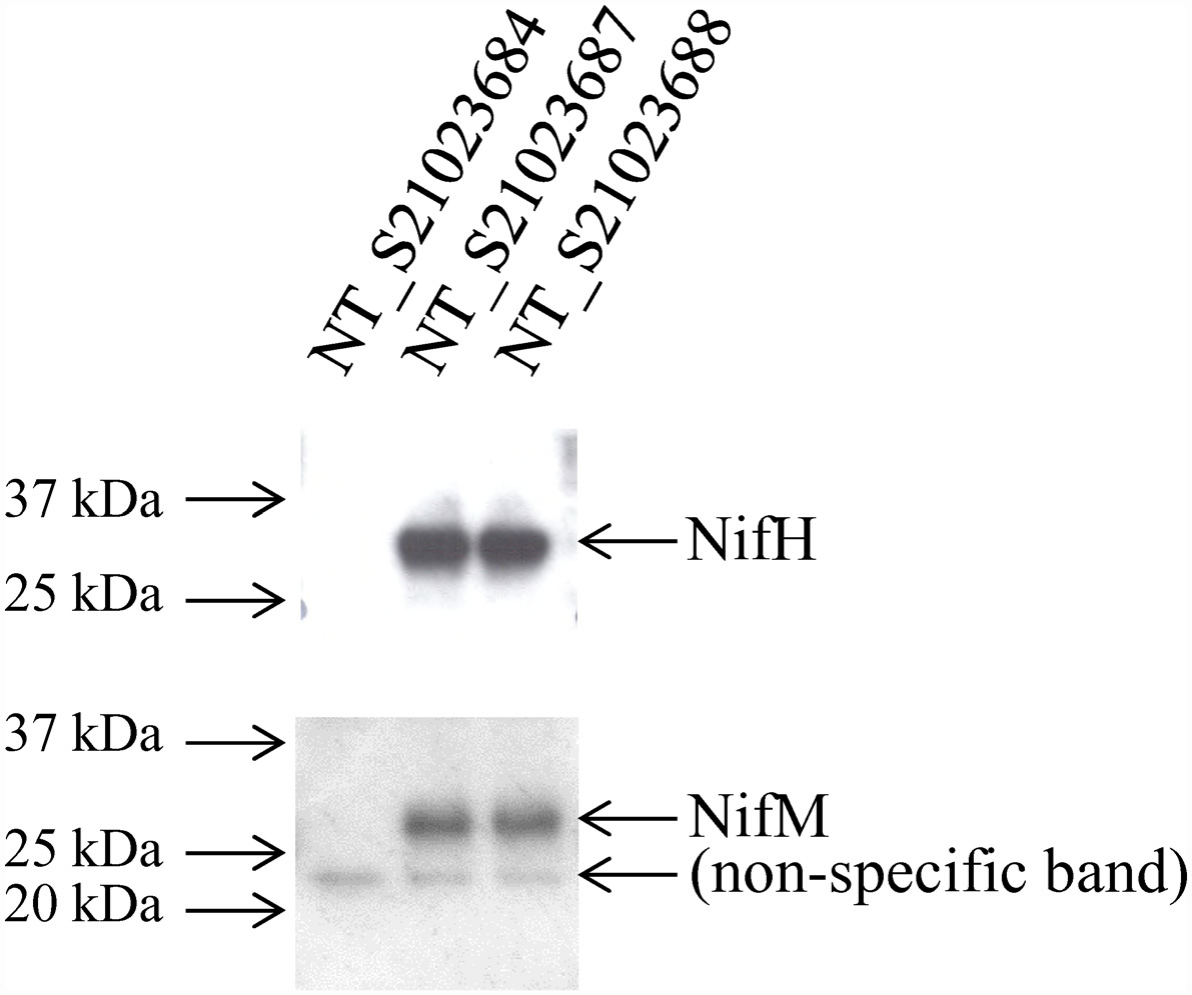
NifH and NifM proteins are expressed in transplastomic lines NT_S21023687 and NT_S2103688, but not in the control line NT_S21023684. Total protein extracts were analyzed by western blot analysis to detect proteins that were immunoreactive to antibodies raised against either the NifH (31 kDa) or NifM proteins (33 kDa). Lane 1, NT_S21023684 (negative control); lane 2, NT_S21023687; lane 3, NT_S21023688. A weak non-specific band (22 kDa) was detected with antibodies raised against NifM protein in all total protein extracts analyzed in this experiment. Select bands of molecular weight marker (Precision Plus Protein Dual Color Standards, Bio-Rad) are shown.

To demonstrate the production of the NifH and NifM proteins, protein fractions from leaf tissue of transplastomic lines were analyzed via western blot analysis using antibodies raised against either the NifH or NifM proteins. Fig 2 shows results of the analysis, where lanes 2 and 3 (lines NT_S21023687 and NT_S21023688) contained proteins that produced immunoreactive bands of the expected size for the NifH and NifM proteins. The line NT_S21023684 (lane 1) that resulted from transformation of the vector lacking *nif* genes did not demonstrate these immunoreactive bands. These results demonstrated that NifH and NifM proteins were produced from transgenes integrated into the plastid genome.

### *In vitro* activity of plant-produced Fe protein

**Fig 3:**
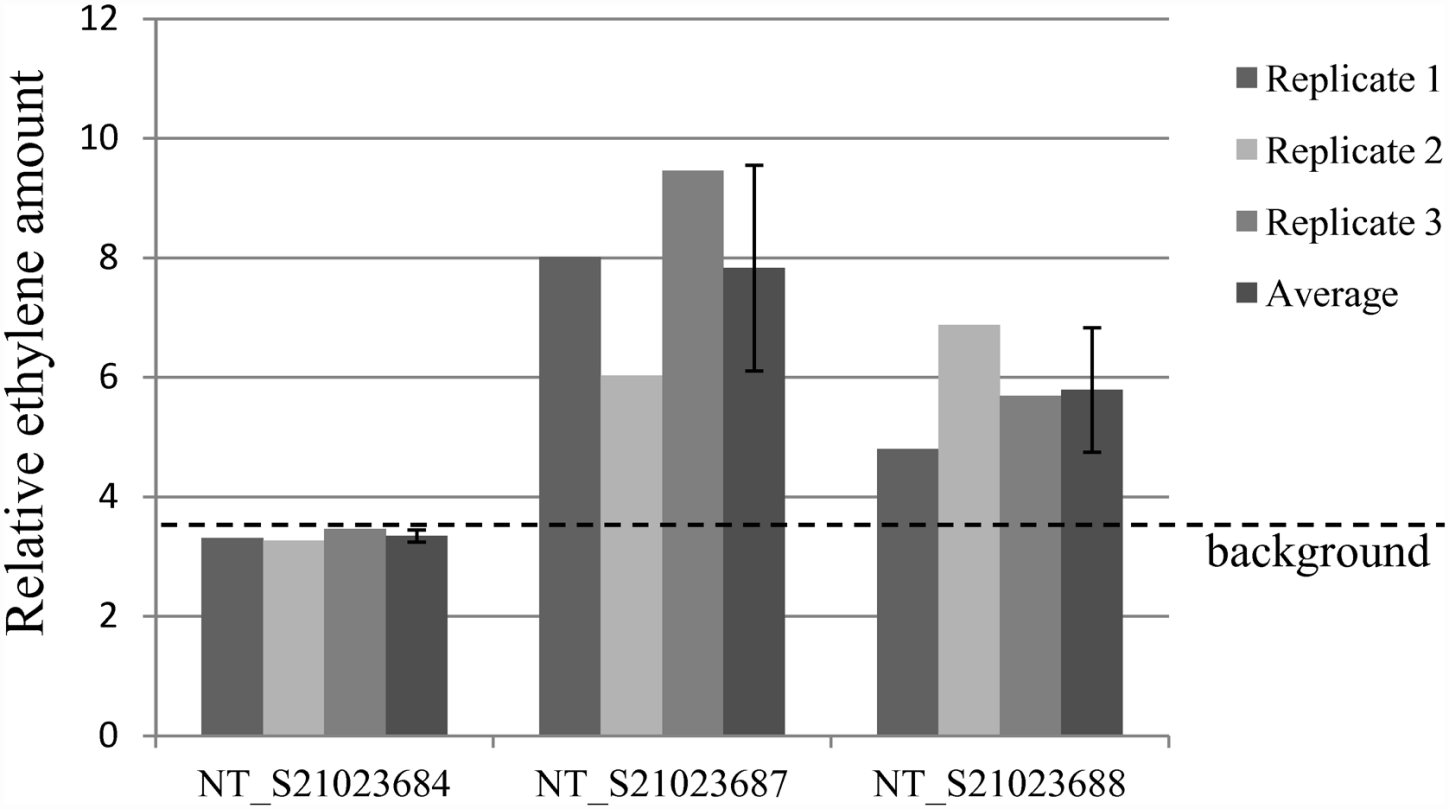
Transplastomic plants expressing NifH and NifM proteins produce active Fe subunit of nitrogenase. Fe-protein enriched fractions from transplastomic lines and corresponding fractions from control plants were combined with bacteria-produced MoFe subunit. The enzymatic activity was evaluated via the acetylene reduction assay (see Materials section). Relative ethylene amounts produced after 3 hour incubations are shown. The experiment was repeated three times using three independent biological replicates (results for which are shown as Replicates 1–3), and error bars represent standard deviation of these three experiments done on three different days. Because ethylene is a minor contaminant in acetylene preparations [45], ethylene level present in the negative control samples (NT_S21023684 line derived from the transformation of pMON253685 which lacked *nif* genes) was used as the background level and is indicated with a dashed line.

To evaluate the presence of the enzymatic activity of the plant-produced Fe protein of nitrogenase, protein fractions were generated from transgenic plants incubated under ambient room light (10 μmol/m^2^s) and low oxygen conditions (10% O_2_). Leaf tissue from the plants was excised, and Fe-protein enriched fractions were generated following a modified chloroplast-enrichment procedure[35, 36]. The fractions containing the plant-produced Fe protein were incubated with the active MoFe protein purified from *A*. *vinelandii*, and the enzymatic activity was evaluated *in vitro* via the acetylene reduction assay (Fig 3). Protein fractions from the transplastomic lines NT_S21023687 and NT_S21023688 demonstrated production of ethylene that was above the background level present in samples from plants lacking *nif* genes (line NT_ S21023684). Without the addition of the bacteria-produced MoFe subunit, the protein fractions did not demonstrate enzymatic activity (data not shown), indicating that the increased levels of ethylene in the transplastomic samples were specific to Nif proteins and not due to increased levels of the ethylene production in the transgenic plants. These results demonstrated that expression of NifH and NifM proteins in transplastomic plants resulted in detectable Fe protein activity as shown by production of ethylene levels beyond the background level present in samples lacking *nif* genes.

## Discussion

Here we report the production of the enzymatically-active, plant-produced Fe protein of nitrogenase from transgenic plants. The results suggest that it should be possible to produce an active nitrogenase enzyme in plants and, therefore, enable plants to synthesize their own nitrogen fertilizer. The transgenic lines described here have the potential to serve as a model system that could improve our understanding of current limitations of nitrogenase production in plants and point us to optimization approaches needed for the production of functional nitrogenase able to support plant metabolism in the absence of an externally supplied nitrogen fertilizer. The transplastomic lines described here might serve as a unique starting point for further engineering of the numerous genes required for *in planta* expression of fully functional nitrogenase. For example, retransformation of NT_S21023687 and NT_S21023688 lines with either additional transplastomic transgenes or nuclear-encoded plastid-targeted transgenes could be attempted to express the functional nitrogenase.

The oxygen sensitivity of nitrogenase still remains a significant obstacle to overcome, particularly in producing commercially useful levels of nitrogen fixation. Our results indicate that, when transgenic plants are incubated at low oxygen concentrations (10% oxygen, which is half of the oxygen concentration in the air), reproducible activity of the Fe subunit can be detected (Fig 3). However, our preliminary results indicate that when the plants were incubated in the ambient air, no significant enzymatic activity was detected, even though the Nif proteins were still expressed in the leaf tissue (data not shown). This suggested that an additional optimization focused on decreasing inter-cellular oxygen levels (such as targeting the production of Nif proteins to non-photosynthetic tissues, e.g. root) may be required for engineering of nitrogen-fixing plants. Our preliminary analysis indicated that in the transplastomic lines described here, *nifH* and *nifM* were expressed at very low level in the root tissue, supporting previously published results that the promoter and 5’ leader combination (*Prrn*:*G10L*) used in this study provided a low level of transplastomic expression in root tissue[37], and suggesting that additional optimization of expression elements might be needed for transplastomic production of Nif proteins in root tissue.

The detected enzymatic activity of plant-produced Fe subunit was reproducible, but relatively low (as compared to relative activity of similar amounts of purified bacteria-produced Fe protein). The plant protein fractions used in the enzymatic activity assay were essentially enriched chloroplast fractions[35] and therefore are expected to contain a variety of cell membranes, small molecules and proteins, including ATP-consuming enzymes, all of which could potentially inhibit enzymatic activity. Our preliminary data indicated that the addition of similar protein fractions from wild type plants significantly inhibited activity of bacteria-produced nitrogenase, suggesting that the plant protein fractions interfered with *in vitro* activity assay (N. Ivleva, unpublished). Western blot analysis indicated that the decrease in the activity levels in these samples was not caused by degradation of the Fe subunit. The results suggested that, if further enriched, the relative activity of the plant-produced Fe subunit might be higher than shown in Fig 3. Further purification of the plant-produced Fe protein will be needed to evaluate the specific activity of the protein, as previously it was reported that *in vitro* activity of nitrogenase is highly dependent on the relative purity of the enzyme[38].

The transgenic plants did not show any phenotypic differences when compared to the wild type plants (e.g. in germination rate, growth rate or plant appearance), indicating that production of transgenic NifH and NifM did not significantly affect plant metabolism or development. Previously, it was suggested that expression of fully functional nitrogenase could significantly affect energy balance of plants[13]. Our finding suggests that at least the expression of one of the two subunits does not appear to affect transgenic plant growth.

The presented results describe transplastomic production of the Fe subunit in which *nif* genes are expressed from the plastid genome. Our initial experiments were conducted using a transient expression system in which tobacco plants were infiltrated with *Agrobacterium* strains containing vectors that produced chloroplast-targeted Nif proteins. The produced protein samples yielded activity levels similar to those of transplastomic plants, suggesting that it is possible to produce Nif proteins in the cytoplasm and then export them to organelles (W. Donovan, N. Ivleva, unpublished). This suggests that it is possible to produce the active enzyme from *nif* genes engineered into the nuclear genome, and, in particular, to target them to mitochondria. At the same time the possibility of expressing *nif* genes from the plastid genome as described here might provide a unique set of advantages, including a possibility of high levels of transgenic expression, simultaneous production of multiple transgenic transcripts via operon expression, and improved folding of transgenic *nif* proteins due to presence of bacterial-like chaperons[39–41]. The main challenge to produce active nitrogenase from the plastid genome is the enzyme’s proximity to the source of oxygen production within the plant cell, though a possibility of nitrogenase expression in non-photosynthetic plastids could improve the stability of the transgenic enzyme[9]. In the future, *nif* transgenes may be expressed in tissues with limited levels of oxygen, such as in non-green tissues of roots, or only during the night, when oxygen production is reduced, thus helping to ensure the stability of the Nif complex. The current lack of a robust plastid transformation procedure for monocots (including maize) could significantly delay development of nitrogen-fixing crops via transgene integration into the plastid genome of these plants; therefore, a nuclear localization of the transgenes should also be considered a viable pathway for development of nitrogen-fixing crops.

Despite the limitations described above, this report represents the first successful step towards engineering biological nitrogen fixation in plants. The report identifies key areas crucial for further development. Furthermore, the generated tobacco lines represent tools for transgenic expression of *nif* genes and will help the scientific community to identify other rate limiting steps for development of fully functional nitrogen-fixing plants. These plants can be used as key starting materials for additional transgenic engineering in either the chloroplast or nuclear compartment.

## Methods

### Expression vectors and plastid transformation

The plastid transformation vectors pMON253685 and its derivative pMON261406 were designed to integrate transgenic sequences into the tobacco plastid genome between the resident *rbcL* and *accD* genes of the tobacco plastid genome. To engineer pMON253685, a DNA sequence corresponding to positions 56,494–60,959 of the tobacco chloroplast genome (GenBank accession Z00044.2) (encompassing the *atpB*, *rbcL* and *accD* gene region) was synthesized and subsequently cloned (Fig 1B). For insertion of transgenes, several restriction enzyme sites for cloning were created downstream of the *rbcL* coding region. An *SbfI* site was created by changing nucleotides TAATT to GCAGG corresponding to positions 59,446–59,450 of the tobacco chloroplast genome, a *SwaI* site was created by insertion of a T at position 59,469 and a *PmeI* site was created by insertion of TAAAC at position 59,485. A chimeric *aadA* gene driven by plastid *psbA* gene 5’- and 3’-end regulatory sequences[33, 42] (GenBank accession # EF416277) was inserted into the *SwaI* and *PmeI* sites to create pMON253685.

The *SbfI* and *SwaI* sites were used for cloning of the *nif* operon cassette (Fig 1A) resulting in pMON261406. DNA sequences encoding *nifH* and *nifM* protein coding regions from *Azotobacter vinelandii* genome (GenBank accession # CP005095) were expressed in pMON261406 from the ribosomal RNA promoter *Prrn* (position 102565–102682, GenBank accession #Z00044.2)[32], and efficient translation of both transgenes was conferred by the bacteriophage T7 gene 10 leader *G10L*[32]. To stabilize transcripts, 3’-ends from tobacco plastid genes *rps16*[33] and *petD* (J. Staub, unpublished) were used[33] (position 4942–5090 and 80271–80462,correspondingly; GenBank accession #Z00044.2). The contiguous sequence of the *nif* operon cassette has been entered into GenBank (accession # KX650081). The resultant transformation vectors (pMON253685 and pMON261406) carried 3 kb left flanking region (*SacI-SbfI* fragment, Fig 1B) and 1.5 kb right flanking region (*PmeI-SphI* fragment, Fig 1B) to direct integration of the transgenes into the plastid genome by homologous recombination.

To introduce the pMON253685 and pMON261406 into tobacco, the protocol for chloroplast transformation was followed as described earlier[30, 43]. Fully expanded tobacco leaves (*Nicotiana tabacum cv*. Petit Havana) were harvested and bombarded with gold particles coated with DNA (pMON253685 or pMON261406) using a biolistic particle delivery system (PDS-1000/He; Bio-Rad, Hercules, CA). The bombarded leaves were placed on a selective media containing spectinomycin at 500 mg/L. Spectinomycin resistant transformants were identified, and their shoots were placed on the selective media for one more round of selection to ensure homoplasmy of the transformants. The resulting shoots were rooted and transitioned to soil, and their leaf tissue was analyzed to confirm homoplasmy.

### Plant Growth

Tobacco plants (*Nicotiana tabacum* cv. Petit Havana) were grown in a growth chamber under the following conditions: 25°C/23°C day/night temperature, 16 hour photoperiod, light intensity of 550 μmol/m^2^s, 70% humidity. Plants were grown in Sun Gro MVP (MM200) soil and sub-irrigated every morning. A fertilizer (Peters 20-20-20) was added every other day. Prior to protein extraction, three-week-old plants were moved to an anaerobic chamber (Vinyl Hypoxic Glove Box, Coy Laboratory Products) and incubated for 3 days under reduced oxygen conditions (10% O_2_, 0.3% CO_2_, balance N_2_). During the incubation in the anaerobic chamber, the plants were kept under ambient room light (10 μmol/m^2^s).

### Southern blot analysis

Genomic DNA was extracted from wild type tobacco plants and transgenic lines using the Qiagen DNEasy Plant Maxi Kit and the Omega Bio-Tek Plant Maxi Kit according to manufacturer’s instructions and quantified on the Nanodrop spectrophotometer. Genomic DNA was digested with *PvuII* or *EcoO109I* and precipitated with ethanol. Digests were loaded on 0.8% agarose gels with DIG labeled markers II and VII (Roche). Gels were run overnight, stained for 30 minutes with SYBR Safe (Invitrogen) and photographed. Gels were blotted for 4 hours using the alkaline transfer method[44] using a DIG nylon N+ membrane (Roche). The DNA was UV crosslinked to the membrane. Probe sequences of the *nifH*, *nifM* and *rbcL* genes were amplified via PCR from pMON261406 (Fig 1C) using oligonucleotide primers (*rbcL* forward primer 5’-AAAGAGTACAAATTGACTTATTATAC-3’ and reverse primer 5’-TTACTTATCCAAAACGTCCACTGCTG-3’; *nifH* forward primer 5’-ATGCGTCAATGCGCCATCTACGGCAA-3’ and reverse primer 5’-TCAGACTTCTTCGGCGGTTTTGCCGA-3’; *nifM* forward primer 5’-ATGGCATCTGAGCGTCTCGCCGACG-3’ and reverse primer 5’-TTATCCATGGGCGAGGTTCTCCAAAG-3’). The probes were labeled using the Roche DIG Probe Synthesis Kit. The DIG procedure was performed according to the manufacturer’s recommendations.

### Protein extraction and Western blot analysis

To maintain anaerobic conditions during protein extraction from leaf tissue, samples and buffers were kept either inside an anaerobic chamber maintained at 0% oxygen or (during centrifugation) inside sealed centrifuge tubes. Samples and buffers were kept at 4°C throughout the preparation. Prior to use, all buffers were flushed with argon to remove oxygen from the solutions. The Fe-protein enriched fractions were generated from tobacco leaves following the chloroplast-enrichment procedure originally described for *Arabidopsis thaliana* [35] with some modifications[36]. For each sample, 4 grams of leaf tissue were harvested from 3 transgenic plants incubated at 10% oxygen. The tissue was gently ground for 3 minutes in the presence of freshly prepared 20 ml of D-MOPS buffer (400 mM D-mannitol, 25 mM MOPS, 10 mM tricine, 1% PVP-40, 0.1% BSA, pH 7.5, 0.5% Protease Inhibitor Cocktail (Sigma, P8340), 5 mM dithionite). The homogenate was filtered through Miracloth (Millipore), resulting in a total protein fraction. The total protein fraction was spun at 100 g for 10 min; the supernatant was collected and spun at 1,000 g for 5 min. The resulting pellet was re-suspended in 0.5 ml of D-MOPS buffer, marked as the Fe-protein enriched fraction, frozen in liquid nitrogen and stored at -80°C until further analysis. The Fe-protein enriched fraction (and a corresponding fraction from plants lacking Nif proteins from NT_S21023684 line) resulted in protein suspension at 11 mg total protein/ml.

To demonstrate the production of the NifH and NifM proteins, the total protein fraction generated from leaf tissue as described above was diluted in Laemmli buffer (BioRad, Hercules, CA) according to manufacturer’s recommendations. For Western blot analysis, protein samples were separated on 4–20% TGX gels (Bio-Rad) using Tris/Glycine/SDS buffer (Bio-Rad). The protein was transferred to 0.2 μm PVDF membranes using Trans-Blot Turbo Transfer System (Bio-Rad). Western blot analysis was conducted using WesternBreeze Immunodetection Kit (Invitrogen) and BenchPro 4100 Processing Station (Invitrogen) per manufacturer’s recommendations. Specific antibodies raised in rabbit against NifH and NifM proteins were used in the analysis at 1:10,000 or 1:1,000 dilution, respectively.

### Activity assay

Frozen Fe-protein enriched fractions were thawed while being flushed with argon. The bacteria-produced MoFe subunit preparation (10 μl at 0.85 μg/ul) was diluted with 550 μl of freshly prepared D-MOPS buffer (see above). One hundred thirty μl of Fe-protein enriched plant-produced fractions were added to the mix. Two hundred ninety μl of ATP regeneration buffer (17 mM MgCl_2_, 8.3 mM ATP, 83 mM creatine phosphate, 0.33 mg/ml creatine phosphokinase, 0.3% Tween-20 in D-MOPS buffer, see above) was added. The assay was conducted in 10 ml glass vials. The vials were sealed with rubber stoppers, flushed with argon, and injected with 1 ml acetylene gas which was prepared freshly by mixing calcium carbide with water. The vials were gently shaken (190 rpm) at 30°C for 3 hours.

The *in vitro* activity was evaluated by estimating the presence of ethylene (the product of the acetylene reduction assay of nitrogenase) after a 3 hour incubation. One ml of gaseous phase from each vial was subjected to gas chromatography analysis to assess the areas of the ethylene peak using Carboxen-1010 PLOT Supelco GC column (Sigma) and Hewlett Packard HP 6890 Series GC system (HP). Ethylene presence was quantified by integrating the 2.8 min peak using Agilent GC ChemStation software (B.01.03 version). The areas of the peaks corresponding to ethylene were calculated and presented as relative ethylene amount produced after 3 hour incubation. Because ethylene is a minor contaminant in acetylene preparations [45], ethylene level present in the negative control samples (NT_S21023684 line derived from the transformation of pMON253685 which lacked *nif* genes) was used as the background level. Similar background levels of ethylene were observed in other samples not expected to produce ethylene such as reactions containing MoFe only or plant-produced fractions only. The experiment was repeated at least three times using independent biological replicates (Fig 3), and error bars represent standard deviation of three experiments done on three different days.
